# Semaphorin 3F (SEMA3F) influences patient survival in esophageal adenocarcinoma

**DOI:** 10.1038/s41598-024-71616-8

**Published:** 2024-09-04

**Authors:** Karl Knipper, Su Ir Lyu, Jin-On Jung, Niklas Alich, Felix C. Popp, Wolfgang Schröder, Hans F. Fuchs, Christiane J. Bruns, Alexander Quaas, Henrik Nienhueser, Thomas Schmidt

**Affiliations:** 1 Faculty of Medicine and University Hospital of Cologne, Department of General, Visceral and Cancer Surgery, University of Cologne, Cologne, Germany; 2 Faculty of Medicine and University Hospital of Cologne, Institute of Pathology, University of Cologne, Cologne, Germany; 3 Department of General, Visceral and Transplant Surgery, University of Heidelberg, Heidelberg, Germany

**Keywords:** Esophageal adenocarcinoma, SEMA3F, Semaphorin, NRP2, Neuropilin, Lymph node metastasis, Tumour biomarkers, Oesophageal cancer

## Abstract

In esophageal adenocarcinoma, the presence of lymph node metastases predicts patients' survival even after curative resection. Currently, there is no highly accurate marker for detecting the presence of lymph node metastasis. The SEMA3F/NRP2 axis was initially characterized in axon guidance and recent evidence has revealed its significant involvement in lymphangiogenesis, angiogenesis, and carcinogenesis. Hence, the objective of this study was to elucidate the roles of SEMA3F and its receptor NRP2 in esophageal adenocarcinoma. We conducted an immunohistochemical evaluation of SEMA3F and NRP2 protein expression in 776 patients with esophageal adenocarcinoma who underwent Ivor-Lewis esophagectomy at the University Hospital of Cologne. Total and positive cancer cell counts were digitally analyzed using QuPath and verified by experienced pathologists to ensure accuracy. Positive expression was determined as a cell percentage exceeding the 50th percentile threshold. In our cohort, patients exhibiting SEMA3F positive expression experience significantly lower pT- and pN-stages. In contrast, positive NRP2 expression is associated with the presence of lymph node metastases. Survival analyses showed that the expression status of NRP2 had no impact on patient survival. However, SEMA3F positivity was associated with a favorable patient survival outcome (median OS: 38.9 vs. 26.5 months). Furthermore, SEMA3F could be confirmed as an independent factor for better patient survival in patients with early tumor stage (pT1N0-3: HR = 0.505, *p* = 0.014, pT1-4N0: HR = 0.664, *p* = 0.024, pT1N0: HR = 0.483, *p* = 0.040). In summary, SEMA3F emerges as an independent predictor for a favorable prognosis in patients with early-stage esophageal adenocarcinoma. Additionally, NRP2 expression is linked to a higher risk of lymph node metastases occurrence. We hypothesize that low SEMA3F expression could identify patients with early-stage tumors who might benefit from more aggressive treatment options or intensified follow-up. Furthermore, SEMA3F and its associated pathways should be explored as potential tumor-suppressing agents.

## Introduction

The prognosis for patients with esophageal adenocarcinoma remains bleak despite extensive research efforts and multimodal therapy regimens, often accompanied by high morbidity rates^1^. Lymph node status is recognized as a critical prognostic indicator for patients with esophageal adenocarcinoma^2^. In this context, not only the number of metastasized lymph nodes is pivotal, but also the total number of lymph nodes resected and the lymph node ratio (the proportion of metastasized to resected lymph nodes) are significant predictors of patient survival^3,4^. Furthermore, the lymph node status at the initial staging serves as a parameter for clinical decision-making. Patients with low cT-stage and negative cN-stage qualify for sole endoscopic resection or primary tumor resection^5^. In endoscopic resection patients do not undergo node resection. Therefore, an accurate assessment of the lymph node status during staging is of a crucial importance. It must also be noted that patients with a low T-stage can still have metastasized lymph nodes^6^. Consequently, there is a pressing need for a biomarker capable of predicting lymph node status and additionally identifying high-risk individuals within patient groups typically considered low-risk.

Semaphorins are a family of proteins that were first described as important molecules for axonal guidance during neural development^7^. Since then, numerous subtypes have been identified across various organisms, including vertebrates, invertebrates, and viruses^7^. Semaphorin 3 refers to secreted molecules that bind to their receptors, neuropilin^7,8^.

Semaphorins have been detected in the development of various organs such as the parathyroid, thymus, and many others^9,10^. Furthermore, semaphorins have been implicated not only in physiological mechanisms but also in influencing cell proliferation, angiogenesis, and the development of metastases in cancer^11^. Semaphorin 3F (SEMA3F) plays a complex role in cancer progression that is not yet fully understood. Transcriptome profiling of hepatocellular carcinomas suggests that SEMA3F may promote metastasis development by activating focal adhesion pathways^12^. SEMA3F acts mainly through its receptor Neuropilin 2 (NRP2)^13^. Conversely, SEMA3F is characterized as a tumor suppressor gene in esophageal squamous cell carcinoma, where low mRNA expression levels of SEMA3F are associated with poor survival outcomes and a positive lymph node status. Furthermore, the SEMA3F expression correlated negatively with the NRP2 expression^14^. However, lymph node metastatic patterns seem to differ between esophageal adenocarcinoma and esophageal squamous cell carcinoma^15^. The role of SEMA3F and NRP2 in esophageal adenocarcinoma remains unclear. Consequently, the objective of this study was to assess NRP2 and its ligand SEMA3F as potential biomarkers for patient survival and their ability to predict lymph node metastases in esophageal adenocarcinoma.

## Materials and methods

### Patients and tumor samples

In this study, 776 patients were included. The inclusion criteria were esophageal adenocarcinoma, and Ivor-Lewis esophagectomy conducted with curative intention. Patients with survival of less than 30 days after surgery or insufficient tumor sample quality were excluded. All patients underwent surgery from 1998 until 2019 at the University Hospital of Cologne. Written informed consent was obtained from every patient. The study was approved by the Ethics Committee of the University Hospital of Cologne (ethics committee number: 21-1146) and was conducted in accordance with the declaration of Helsinki. The specimens’ pathological tumor ((y)pT) as well as lymph node status (following neoadjuvant therapy) ((y)pN), lymphatic invasion (L), vascular invasion (V), perineural invasion (Pn), and grading (G) were analyzed according to the 7th edition of the Union for International Cancer Control at our department of pathology. Tissue cylinders (1.2 mm) were punched out of each tumor sample with a semi-automated precision instrument and transferred to a paraffin-embedded tissue microarray (TMA). Stainings were conducted on 4 µm thick slices.

### Immunohistochemistry

Immunohistochemical stainings and control stainings were performed according to the manufacturers' advice. Stainings were done for SEMA3F (HPA035008, Sigma-Aldrich, St. Louis, USA) and NRP2 (HPA039980, Sigma-Aldrich, St. Louis, USA). The automatic staining system Leica BOND-MAX (Leica Biosystems, Wetzlar, Germany) was used for all stainings. The stained slides were digitalized using Aperio GT 450 DX (Leica Biosystems, Wetzlar, Germany). The stainings were analyzed digitally with QuPath v0.3.2 as described before (Suppl. Fig. 1)^16^. The percentage of positive cells in tumor tissue was calculated using a positive cell detector and tissue classifier. Quality control was performed by two experienced pathologists (S.L. and A.Q.). The total study cohort was split up into two cohorts: negative and positive marker expression. Positivity was defined as values above the 50th percentile.

### Statistical analysis

Statistical analyses were performed using IBM SPSS Statistics (Version 28.0.1.1). *p* values below 0.05 were considered statistically significant. Evaluation of qualitative value was performed with the Chi-Square test. Survival analyses were made with Kaplan-Meier curves and the log-rank test. Univariate and multivariate Cox regression analyses were used to detect interdependencies of clinicopathologic and survival data. Only clinicopathologic values, which received *p* values below 0.20 in univariate Cox regression analyses, were included in multivariate Cox regression analyses. All clinical data was collected prospectively and analyzed retrospectively. Overall survival was measured from date of surgery until death or loss of follow-up.

## Results

All 776 included patients underwent Ivor-Lewis esophagectomy with curative intention. Clinicopathological values are depicted in Table 1. Of these patients, 685 (88.3%) were male. 70.6% (n = 548) of the included patients received perioperative/neoadjuvant therapy prior to resection. Lymph node metastases were detected in 458 patients (59.0%). SEMA3F and NRP2 immunohistochemical stainings were performed and analyzed digitally (Suppl. Fig. 1). The cohort was divided into two groups according to the negative or positive marker expression. SEMA3F-positive patients showed significantly lower pT-stages, lower pN-stages, fewer lymphovascular and perineural invasions (p(T) = 0.016, p(N) = 0.041, p(L) = 0.026, p(Pn) = 0.033, Table 1). On the contrary, patients with positive NRP2-stainings showed significantly more lymph node metastases, and more often lymphovascular invasion (p(N) = 0.041, p(L) = 0.002, Table 1).

**Table 1:** General clinicopathological values of the total study population and patients with negative or positive SEMA3F as well as NRP2 marker expression.

Characteristic	Total	SEMA3F		*p* value	NRP2		*p* value
		Negative	Positive		Negative	Positive	
	n (%)	n (%)	n (%)		n (%)	n (%)	
No. of patients	776 (100)	388 (100)	388 (100)		388 (100)	388 (100)	
Sex				0.147			0.220
Male	685 (88.3)	336 (86.6)	349 (89.9)		348 (89.7)	337 (86.9)	
Female	91 (11.7)	52 (13.4)	39 (10.1)		40 (10.3)	51 (13.1)	
Age				0.219			0.718
< 65	433 (55.8)	208 (53.6)	225 (58.0)		214 (55.2)	219 (56.4)	
≥ 65	343 (44.2)	180 (46.4)	163 (42.0)		174 (44.8)	169 (43.6)	
Median overall survival (months)	31.6	26.5	38.9		30.6	32.1	
(95% confidence interval)	(26.9–36.6)	(22.3–30.7)	(24.5–53.4)		(24.7–36.5)	(24.8–39.4)	
Perioperative/neoadjuvant therapy				0.753			0.636
No	228 (29.4)	112 (28.9)	116 (29.9)		111 (28.6)	117 (30.2)	
Yes	548 (70.6)	276 (71.1)	272 (70.1)		277 (71.4)	271 (69.8)	
Perioperative/neoadjuvant				**0.050**			**0.004**
treatment regime							
CROSS	288 (29.6)	140 (36.1)	148 (38.1)		164 (42.3)	124 (32.0)	
FLOT	92 (11.9)	38 (9.8)	54 (13.9)		46 (11.9)	46 (11.9)	
Other	166 (21.4)	97 (25.0)	69 (17.8)		65 (16.8)	101 (26.0)	
None	230 (29.6)	113 (29.1)	117 (30.2)		113 (29.1)	117 (30.2	
(y)pT				**0.016**			0.264
1	143 (18.4)	56 (14.4)	87 (22.4)		68 (17.5)	75 (19.3)	
2	128 (16.5)	70 (18.0)	58 (14.9)		60 (15.5)	68 (17.5)	
3	482 (62.1)	247 (63.7)	235 (60.6)		252 (64.9)	230 (59.3)	
4	23 (3.0)	15 (3.9)	8 (2.1)		8 (2.1)	15 (3.9)	
(y)pN				**0.041**			**0.041**
0	318 (41.0)	145 (37.4)	173 (44.6)		173 (44.6)	145 (37.4)	
1–3	458 (28.7)	243 (62.6)	215 (55.4)		215 (55.4)	243 (62.6)	
L				**0.026**			**0.002**
0	363 (46.8)	166 (42.8)	197 (50.8)		203 (52.3)	160 (41.2)	
1	413 (53.2)	222 (57.2)	191 (49.2)		185 (47.7)	228 (58.8)	
V				0.110			0.161
0	583 (75.1)	290 (74.7)	293 (75.5)		303 (78.1)	280 (72.2)	
1	77 (9.9)	32 (8.3)	45 (11.6)		34 (8.8)	43 (11.1)	
2	116 (14.9)	66 (17.0)	50 (12.9)		51 (13.1)	65 (16.8)	
Pn				**0.033**			0.258
0	508 (65.5)	259 (66.8)	249 (64.2)		259 (66.8)	249 (64.2)	
1	156 (20.1)	65 (16.8)	91 (23.5)		81 (20.9)	75 (19.3)	
2	112 (14.4)	64 (16.5)	48 (12.4)		48 (12.4)	64 (16.5)	
G				0.995			0.762
1	2 (0.3)	1 (0.3)	1 (0.3)		1 (0.3)	1 (0.3)	
2	118 (15.2)	58 (14.9)	60 (15.5)		56 (14.4)	62 (16.0)	
3	103 (13.3)	50 (12.9)	53 (13.7)		54 (13.9)	49 (12.6)	
4	0 (0.0)	0 (0.0)	0 (0.0)		0 (0.0)	0 (0.0)	
Not applicable/unknown	553 (71.2)	279 (71.9)	274 (70.6)		277 (71.3)	276 (71.1)	

Bold print marks *p* values below 0.05.

*CROSS Chemoradiotherapy for Oesophageal Cancer followed by Surgery Study, FLOT fluorouracil, leucovorin, oxaliplatin and docetaxel,* *NRP2* neuropilin 2, *SEMA3F* semaphorine 3F.

**Fig. 1:**
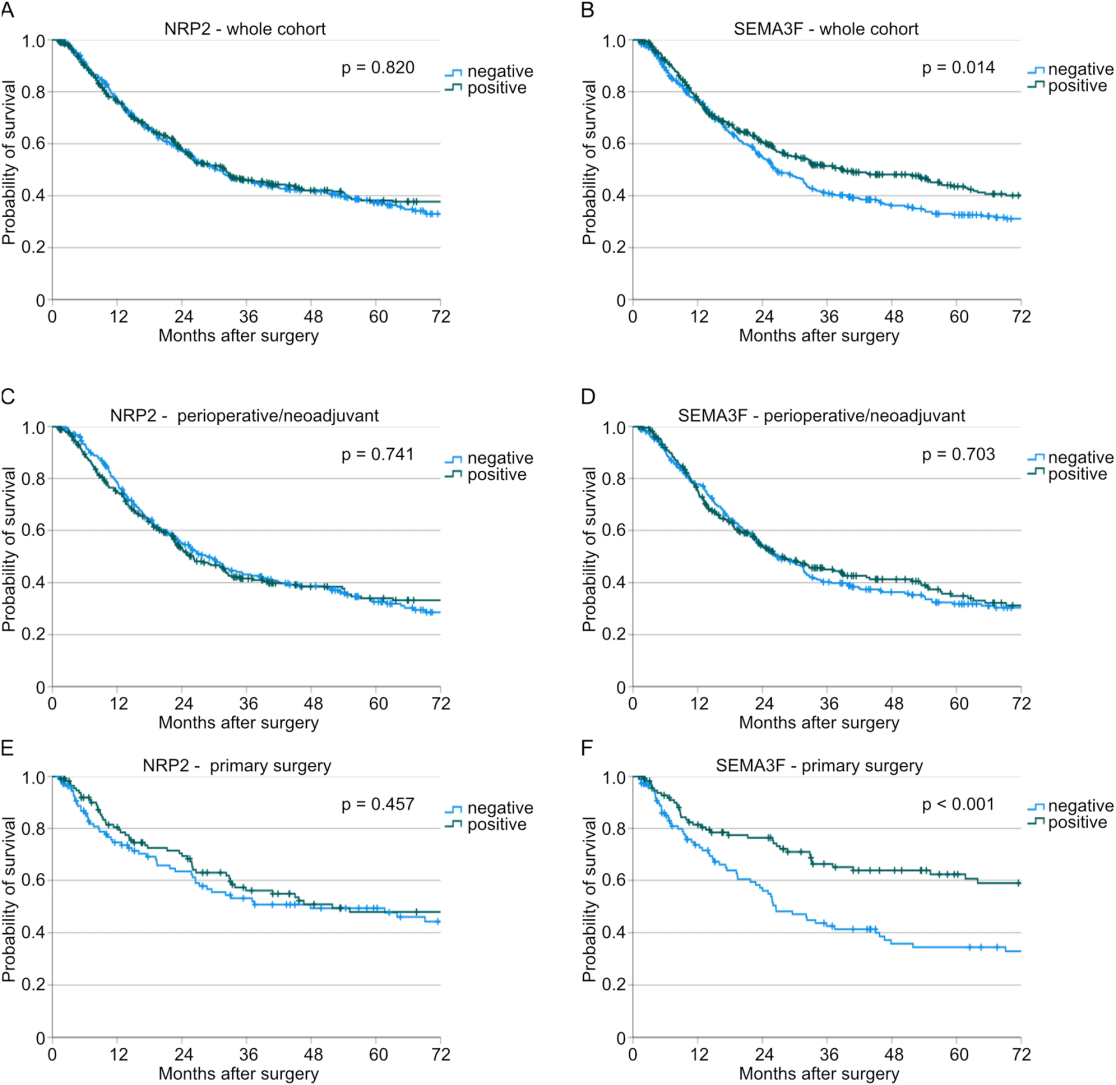
Kaplan-Meier curves for overall survival of the total cohort depending on (**A**) NRP2 expression (n(negative) = 388, n(positive) = 388, *p* = 0.820) and (**B**) SEMA3F expression (n(negative) = 388, n(positive) = 388, *p* = 0.014). Furthermore, Kaplan-Meier curves for overall survival for the perioperative/neoadjuvant treated subgroup depending on (**C**) NRP2 expression (n(negative) = 277, n(positive) = 271, *p* = 0.741) and (**D**) SEMA3F expression (n(negative) = 276, n(positive) = 272, *p* = 0.703) as well as for the primary resected subgroup depending on (**E**) NRP2 expression (n(negative) = 111, n(positive) = 117, *p* = 0.457) and (**F**) SEMA3F expression (n(negative) = 112, n(positive) = 116, *p* < 0.001).

To evaluate the impact of SEMA3F and NRP2 expression on patients' survival, we carried out survival analyses. Here, we could not find an impact of NRP2 expression on overall survival (*p* = 0.820, Fig. 1A). Positive SEMA3F expression correlated with favorable patient survival (38.9 months vs. 26.5 months, *p* = 0.014, Fig. 1B). Since perioperative/neoadjuvant therapy could influence the proteome of tumors, we conducted our survival analyses depending on perioperative/neoadjuvant therapy or primary surgery. NRP2 expression proved to have no impact on patients' survival in both subgroups (p(perioperative/neoadjuvant) = 0.741, p(primary surgery) = 0.457, Fig. 1C and E). SEMA3F showed also no impact in patients after perioperative/neoadjuvant therapy (p = 0.703, Fig. 1D). However, patients, who received primary surgery and showed a positive SEMA3F expression, showed a significantly better overall survival compared to a negative expression (140.9 vs. 26.5 months, *p* < 0.001, Fig. 1F).

Additionally, we conducted survival analyses in a patient subgroup, that received adjuvant therapy due to the perioperative FLOT treatment regime. Here, no significant survival difference could be detected depending on the SEMA3F or NRP2 expression (p(NRP2) = 0.737, p(SEMA3F) = 0.367). In line, patients, who received no adjuvant therapy as a primary therapy regime, also did not show a significant survival difference depending on the SEMA3F or NRP2 expression (p(NRP2) = 0.333, p(SEMA3F) = 0.065).

Following these analyses, we performed multivariate Cox regression analyses for the total cohort and the primary resected subgroup. Here, SEMA3F expression did not prove to be an independent factor for patients' survival (total cohort: HR = 0.785, 95% CI 0.555–1.111, *p* = 0.172, Table 2; primary surgery: HR = 0.747, 95% CI 0.498–1.121, *p* = 0.159, Table 3). Higher age, (y) pT-stage, (y) pN-stage, and lymphovascular invasion were shown to be independent risk factors for worse survival in our cohort (Tables 2 and 3). Female sex and vascular invasion are significant factors for favorable survival (Table 2).

**Table 2:** Multivariate Cox regression analyses of the total study cohort.

Characteristic	Borders	Hazard ratio	95% confidence interval	*p* value
Sex	Female vs male	0.469	0.261–0.841	**0.011**
Age	≥ 65 vs < 65	1.473	1.011–2.146	**0.044**
Perioperative/neoadjuvant therapy	Yes vs no	1.199	0.768–1.874	0.425
(y)pT	≥ 2 vs 1	1.465	1.129–1.903	**0.004**
(y)pN	≥ 1 vs 0	1.701	1.413–2.047	** < 0.001**
L	1 vs 0	1.951	1.293–2.943	**0.001**
V	≥ 1 vs 0	0.563	0.372–0.773	** < 0.001**
G	≥ 2 vs 1	1.176	0.822–1.683	0.376
SEMA3F	Positive vs negative	0.785	0.555–1.111	0.172

Bold print marks *p* values below 0.05.

*SEMA3F* semaphorine 3F.

**Table 3:** Multivariate Cox regression analyses of the primarily resected subcohort.

Characteristic	Borders	Hazard ratio	95% confidence interval	*p* value
Age	≥ 65 vs < 65	1.411	0.904–2.202	0.129
(y)pT	≥ 2 vs 1	1.554	1.169–2.065	**0.002**
(y)pN	≥ 1 vs 0	1.663	1.348–2.051	** < 0.001**
V	≥ 1 vs 0	0.877	0.677–1.137	0.322
G	≥ 2 vs 1	1.237	0.827–1.849	0.300
SEMA3F	Positive vs negative	0.747	0.498–1.121	0.159

Bold print marks *p* values below 0.05.

*SEMA3F* semaphorine 3F.

**Fig. 2:**
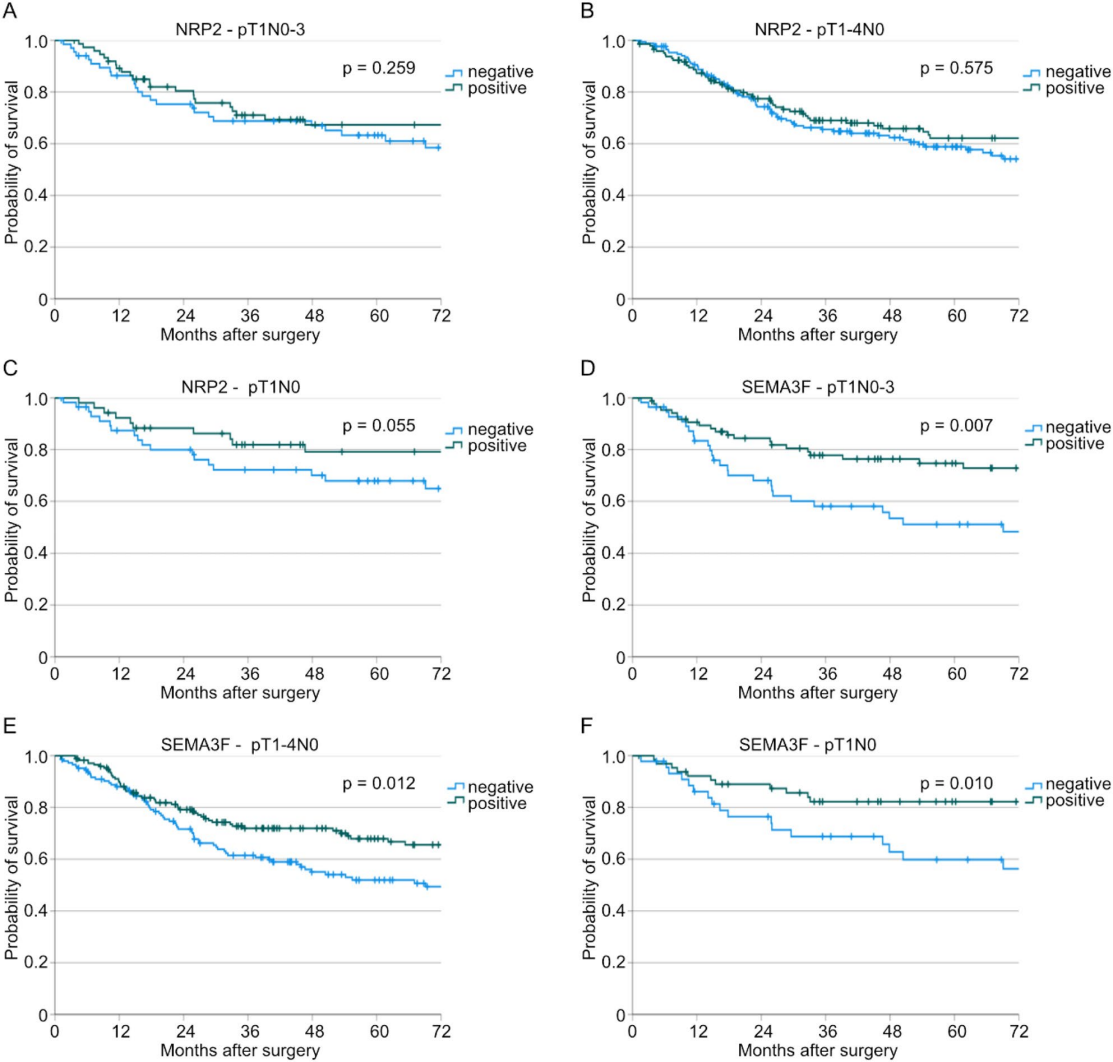
Kaplan-Meier curves for overall survival depending on NRP2 expression in the (**A**) pT1N0-3 (n(negative) = 68, n(positive) = 75, *p* = 0.259), (**B**) pT1-4N0 (n(negative) = 173, n(positive) = 145, *p* = 0.575), and (**C**) pT1N0 subgroup (n(negative) = 58, n(positive) = 52, *p* = 0.055). Furthermore, Kaplan-Meier curves for overall survival depending on SEMA3F expression in the (**D**) pT1N0-3 (n(negative) = 56, n(positive) = 87, *p* = 0.007), (**E**) pT1-4N0 (n(negative) = 145, n(positive) = 173, *p* = 0.012), and (**F**) pT1N0 subgroup (n(negative) = 45, n(positive) = 65, *p* = 0.010).

Since patients with positive SEMA3F expression showed a favorable survival but SEMA3F expression was not an independent risk factor in the total cohort, we carried out further subgroup analyses depending on the (y)pT- and (y)pN-stage (pT1N0-3: Suppl. Table 1, pT1-4N0: Suppl. Table 2, pT1N0: Suppl. Table 3). Here, patients with positive NRP2 expression showed significantly more often lymph node metastases as well as lymphovascular invasion in the subgroup of patients with early (y)pT-stage (p((y)pN) = 0.024, p(L) = 0.019, Suppl. Table 1). No further differences in clinicopathological values depending on the NRP2 or SEMA3F expression could be detected. Additional survival analyses showed no correlation between NRP2 expression and patient survival (p(pT1N0-3) = 0.259, p(pT1-4N0) = 0.575, p(pT1N0) = 0.055, Fig. 2A–C). However, patients with positive SEMA3F expression showed significantly better overall survival compared to patients with negative SEMA3F expression in all three subgroups (p(pT1N0-3) = 0.007, p(pT1-4N0) = 0.012, p(pT1N0) = 0.010, Fig. 2D–F).

Moreover, multivariate Cox regression analyses were carried out. Here, SEMA3F expression could be proven as a significant, independent factor for favorable overall survival in these three subgroups (pT1N0-3: HR = 0.505, 95% CI 0.293–0.870, *p* = 0.014, Suppl. Table 4; pT1-4N0: HR = 0.664, 95% CI 0.465–0.947, *p* = 0.024, Table 4; pT1N0: HR = 0.483, 95% CI 0.242–0.967, *p* = 0.040, Suppl. Table 5). Furthermore, high patient age, perioperative/neoadjuvant therapy, and the occurrence of lymph node metastases were shown to be independent risk factors for worse patient survival (Table 4, Suppl. Tables 4–5).

**Table 4:** Multivariate Cox regression analyses of the pT1-4N0 study cohort.

Characteristic	Borders	Hazard ratio	95% confidence interval	*p* value
Sex	Female vs male	0.611	0.343–1.090	0.095
Age	≥ 65 vs < 65	1.441	1.007–2.063	**0.046**
Perioperative/neoadjuvant therapy	yes vs no	1.494	0.932–2.397	0.096
(y)pT	≥ 2 vs 1	1.207	0.958–1.521	0.111
V	≥ 1 vs 0	0.853	0.680–1.071	0.171
SEMA3F	Positive vs negative	0.664	0.465–0.947	**0.024**

Bold print marks *p* values below 0.05.

*SEMA3F* semaphorine 3F.

In summary, SEMA3F expression could be identified as an independent factor for favorable patient survival in patients with early-stage esophageal adenocarcinoma. Furthermore, positive NRP2 expression was associated with the occurrence of lymph node metastases as well as lymphovascular invasion.

## Discussion

In this study, we investigated the role of SEMA3F and its receptor, NRP2, in patients diagnosed with esophageal adenocarcinoma. We observed a significant correlation between SEMA3F expression and patients' survival. Conversely, there was no observed correlation between NRP2 expression and patient survival in the same cohort. SEMA3F positivity was linked to improved patient survival (median OS: 38.9 months vs. 26.5 months), aligning with similar findings observed in esophageal and oral squamous cell carcinoma cases^14,17^. Furthermore, in-vitro experiments revealed an inhibitory effect of SEMA3F on cell migration and invasion in, for example, oral squamous cell cancer cells or breast cancer cells^18,19^. SEMA3F influences not only the tumor cells directly but also impacts angiogenesis. Here, SEMA3F expression resulted in decreased intratumoral microvessel density in lung cancer models in mice^20^. These effects of SEMA3F are mainly established through the inhibition of mTOR signaling^13^.

Interestingly, divergent roles of SEMA3F are described in various cancer entities. In hepatocellular carcinoma (HCC), a high SEMA3F expression could be correlated with an unfavorable survival^21^. This can be elucidated by the distinct macromolecules measured in the mentioned publications—mRNA versus protein—for SEMA3F. In our study, we measured protein expression using immunohistochemistry.

Additionally, SEMA3F is known to modify the tumor microenvironment^22^. High SEMA3F expression levels are positively correlated with infiltrating B cells, T cells, macrophages, and neutrophils in HCC^21^. The tumor microenvironment consistency is recognized for its variability across different cancer types^23^. Consequently, the influence of SEMA3F may change based on the specific cancer entity and the characteristics of the surrounding tumor microenvironment.

The impact of SEMA3F on patients' survival did not prove to be an independent factor in Cox regression analyses in our total patient cohort or in patients, treated with primary surgery. In subgroup analyses of patients with early tumor stage (pT1N0-3, pT1-4N0, pT1N0), positive SEMA3F expression correlated with better patient survival. Additionally, SEMA3F proves to be an independent factor for a favorable prognosis in these patient subgroups (p(pT1N0-3) = 0.014, p(pT1-4N0) = 0.024, p(pT1N0) = 0.040). Interestingly, SEMA3F does not have an impact on patients' survival in more advanced tumor stages. This could imply an impact on the carcinogenesis of SEMA3F in early tumor stages. Confirming this thesis, SEMA3F expression gets lost during cancer progression in head and neck squamous cell carcinoma (HNSCC)^24^. Inheriting this, SEMA3F negativity could serve as a marker for a high-risk patient cohort in—according to classically used risk definitions—low-risk patient cohorts.

SEMA3F positivity showed a significant correlation with improved survival outcomes in patients who underwent primary surgery, in contrast to those who received multimodal therapy including perioperative/neoadjuvant treatment. This trend was evident both in the overall cohort and among patients diagnosed with early-stage tumors. Additional fundamental studies are required to elucidate the underlying pathway behind this phenomenon.

In our patient cohort, high SEMA3F expression was correlated with a less frequent occurrence of lymph node metastases in esophageal adenocarcinoma (*p* = 0.041). In line with our data, high SEMA3F expression is correlated with a lower risk of occult lymph node metastases in HNSCC^25^. Furthermore, patients with high SEMA3F expressions did not only show fewer lymph node metastases but also less distant metastases in colorectal cancer^26^. Interestingly, we could show that patients with positive NRP2 expression have significantly more often lymph node metastases (*p* = 0.041). Similar effects could be observed in HNSCC^25^. In-vitro analyses showed, that SEMA3F predominantly interacts with lymphatic endothelial cells through its receptor NRP2^24^. Lymphadenectomy is recommended for patients undergoing surgical resection^5^. However, the extensiveness of mediastinal lymphadenectomy correlates with the occurrence of resection-depending morbidity^27^. Clinical trials to minimize morbidity while maximizing diagnostic certainty of complete resection of all tumor cells including lymph nodes are ongoing and recruiting. In the realm of breast cancer and melanoma treatment, the practice of sentinel lymph node resection has demonstrated promise when applied to patients with esophageal cancer^28^. Despite its promising potential, this workflow has not yet been integrated into routine clinical practice and is not recommended by current guidelines^5^. Therefore, biomarkers for the occurrence of lymph node metastases and thus markers for the determination of the individually required extent of lymphadenectomy are needed.

SEMA3F could not only be used as a biomarker for patients' survival but also possibly in therapy planning. For example, the chemosensitivity to fluorouracil of human colorectal cancer cells was investigated in vitro and in vivo. Cells with higher SEMA3F expression showed higher chemosensitivity and a higher rate of cancer cell apoptosis^29^. Moreover, rats injected with lung cancer cells expressing SEMA3F exhibited significantly prolonged survival compared to rats injected with control lung cancer cells^30^. Vice versa the knockdown of SEMA3F reduced the apoptotic activity of rituximab in diffuse large B-cell lymphoma (DLBCLC)^31^. Based on this SEMA3F expression could help to modify the treatment selection.

In summary, our study has identified SEMA3F as a factor associated with favorable patient survival, particularly in those with early-stage esophageal adenocarcinomas. Furthermore, NRP2 expression could be correlated with a higher rate of lymph node metastases. Although retrospective in design, our findings have the potential to greatly inform future clinical decision-making, subject to validation through additional fundamental and prospective clinical studies. Low SEMA3F expression may identify a higher-risk subgroup within the traditionally defined low-risk patient category. This subgroup could benefit from more aggressive treatment options or intensified post-treatment care.

## Conclusions

We demonstrated that positive SEMA3F protein expression is correlated with favorable patient survival in esophageal adenocarcinoma. Moreover, SEMA3F could be identified as an independent factor for better patient survival in patients with early tumor stage (pT1N0-3, pT1-4N0, pT1N0). The SEMA3F receptor NRP2 is associated with a higher likelihood of lymph node metastases. This highlights the necessity for further prospective clinical research to investigate whether negative SEMA3F expression and positive NRP2 expression could identify a subset of patients who are at higher risk. Such markers hold the potential to recalibrate risk assessment in patients who are currently categorized as low-risk by conventional pathological classifications, thereby necessitating more tailored monitoring and management strategies. These patients could potentially benefit from intensified follow-up, more aggressive systematic therapy options, or even extended lymphadenectomy. Additionally, the SEMA3F/NRP2 pathway may present novel therapeutic avenues if subsequent foundational studies corroborate its purported tumor-suppressive capabilities.

## Supplementary Information

Supplementary Information.

## Data Availability

The datasets generated and analyzed during the current study are available from the corresponding author on reasonable request.
