# Supplementary material for: Semaphorin 3F (SEMA3F) influences patient survival in esophageal adenocarcinoma
Source: Sci Rep. 2024 Sep 4;14:20589. doi: 10.1038/s41598-024-71616-8 (PMC11375056; doi:10.1038/s41598-024-71616-8)
Supplement: Supplementary file 1 — Supplementary Information. [file 41598_2024_71616_MOESM1_ESM.docx]

**Supplement Material**

**Semaphorine 3F (SEMA3F) influences patient survival in esophageal adenocarcinoma**

Karl Knipper^1*#^, Su Ir Lyu^2#^, Jin-On Jung^1^, Niklas Alich^3^, Felix C. Popp^1^, Wolfgang Schröder^1^, Hans F. Fuchs^1^, Christiane J. Bruns^1^, Alexander Quaas^2^, Henrik Nienhueser^3^, Thomas Schmidt^1*^

**Supplement Figure 1**

Representative images of (A) immunohistochemical stainings of SEMA3F (left) and NRP2 (right). (B) Exemplary images of the digitally applied positive cell detector (red: positive; blue: negative) as well as (C) the tissue classifier (green: tumor; yellow: stroma; pink: background).


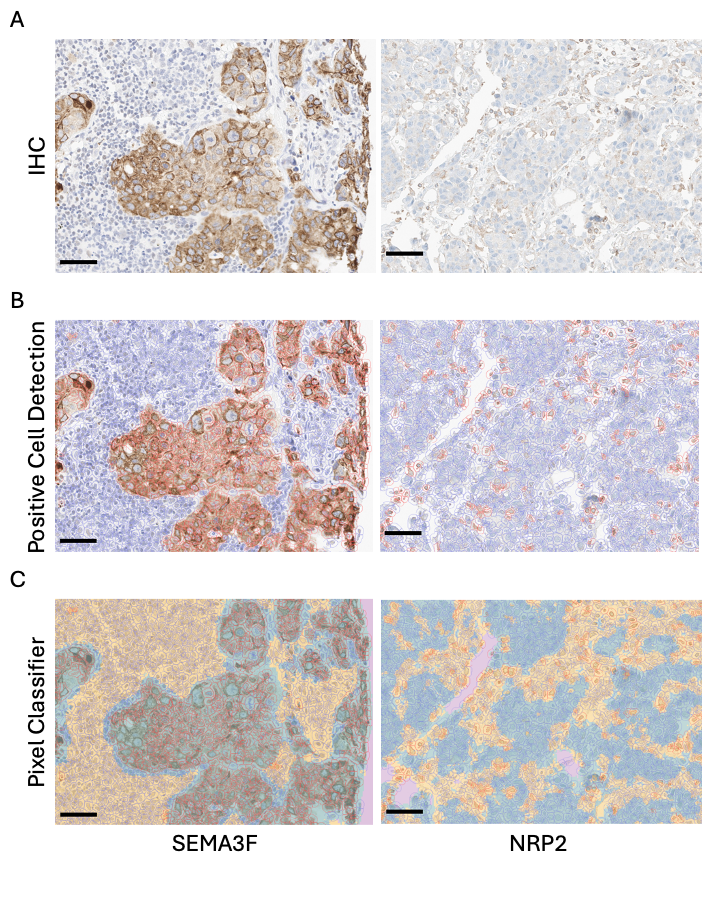


**Supplement Table 1**

General clinicopathological values of patients with pT1N0-3-stage depending on negative or positive SEMA3F as well as NRP2 marker expression. Nr: not reached, NRP2: Neuropilin 2, SEMA3F: Semaphorine 3F. Bold print marks p-values below 0.05.

| **Characteristic** | **Total** | **SEMA3F** | |  | **NRP2** | |  |
| --- | --- | --- | --- | --- | --- | --- | --- |
|  |  | **negative** | **positive** |  | **negative** | **positive** |  |
|  | **n (%)** | **n (%)** | **n (%)** | **p-value** | **n (%)** | **n (%)** | **p-value** |
| **No. of patients** | 143 (100) | 56 (100) | 87 (100) |  | 68 (100) | 75 (100) |  |
| **Sex** |  |  |  | 0.588 |  |  | 0.491 |
| Male | 130 (90.9) | 50 (89.3) | 80 (92.0) |  | 63 (92.6) | 67 (89.3) |  |
| Female | 13 (9.1) | 6 (10.7) | 7 (8.0) |  | 5 (7.4) | 8 (10.7) |  |
| **Age** |  |  |  | 0.198 |  |  | 0.395 |
| < 65 | 81 (56.6) | 28 (50.0) | 53 (60.9) |  | 36 (52.9) | 45 (60.0) |  |
| ≥ 65 | 62 (43.4) | 28 (50.0) | 34 (39.1) |  | 32 (47.1) | 30 (40.0) |  |
| **Median overall survival (months)** | 158.4 | 69.1 | nr |  | 140.9 | nr |  |
| **(95 % confidence interval)** | (88.4-228.4) | (10.5-127.7) | (nr) |  | (57.7-224.1) | (nr) |  |
| **Perioperative/ neoadjuvant therapy** |  |  |  | 0.464 |  |  | 0.500 |
| No | 82 (57.3) | 30 (53.6) | 52 (59.8) |  | 37 (54.4) | 45 (60.0) |  |
| Yes | 61 (42.7) | 26 (46.4) | 35 (40.2) |  | 31 (45.6) | 30 (40.0) |  |
| **(y)pN** |  |  |  | 0.434 |  |  | **0.024** |
| 0 | 110 (76.9) | 45 (80.4) | 65 (74.7) |  | 58 (85.3) | 52 (69.3) |  |
| 1-3 | 33 (23.1) | 11 (19.6) | 22 (25.3) |  | 10 (14.7) | 23 (30.7) |  |
| **L** |  |  |  | 0.952 |  |  | **0.019** |
| 0 | 100 (69.9) | 39 (69.6) | 61 (70.1) |  | 54 (79.4) | 46 (61.3) |  |
| 1 | 43 (30.1) | 17 (30.4) | 26 (29.9) |  | 14 (20.6) | 29 (38.7) |  |
| **V** |  |  |  | 0.653 |  |  | 0.229 |
| 0 | 114 (79.7) | 46 (82.1) | 68 (78.2) |  | 57 (83.8) | 57 (76.0) |  |
| 1 | 1 (0.7) | 0 (0.0) | 1 (1.1) |  | 1 (1.5) | 0 (0.0) |  |
| 2 | 28 (19.6) | 10 (17.9) | 18 (20.7) |  | 10 (14.7) | 18 (24.0) |  |
| **Pn** |  |  |  | 0.891 |  |  | 0.320 |
| 0 | 112 (78.3) | 45 (80.4) | 67 (77.0) |  | 56 (82.4) | 56 (74.7) |  |
| 1 | 3 (2.1) | 1 (1.8) | 2 (2.3) |  | 2 (2.9) | 1 (1.3) |  |
| 2 | 28 (19.6) | 10 (17.9) | 18 (20.7) |  | 10 (14.7) | 18 (24.0) |  |
| **G** |  |  |  | 0.652 |  |  | 0.954 |
| 1 | 2 (1.4) | 1 (1.8) | 1 (1.2) |  | 1 (1.5) | 1 (1.3) |  |
| 2 | 61 (42.7) | 20 (14.9) | 41 (47.1) |  | 28 (41.2) | 33 (44.0) |  |
| 3 | 16 (11.2) | 7 (35.7) | 9 (10.3) |  | 8 (11.8) | 8 (10.7) |  |
| 4 | 0 (0.0) | 0 (0.0) | 0 (0.0) |  | 0 (0.0) | 0 (0.0) |  |
| not applicable/unknown | 64 (44.7) | 28 (50.0) | 36 (41.4) |  | 31 (45.5) | 33 (44.0) |  |

**Supplement Table 2**

General clinicopathological values of patients with pT1-4N0-stage depending on negative or positive SEMA3F as well as NRP2 marker expression. NRP2: Neuropilin 2, SEMA3F: Semaphorine 3F. Bold print marks p-values below 0.05.

| **Characteristic** | **Total** | **SEMA3F** | |  | **NRP2** | |  |
| --- | --- | --- | --- | --- | --- | --- | --- |
|  |  | **negative** | **positive** |  | **negative** | **positive** |  |
|  | **n (%)** | **n (%)** | **n (%)** | **p-value** | **n (%)** | **n (%)** | **p-value** |
| **No. of patients** | 318 (100) | 145 (100) | 173 (100) |  | 173 (100) | 145 (100) |  |
| **Sex** |  |  |  | 0.661 |  |  | 0.439 |
| Male | 277 (87.1) | 125 (86.2) | 152 (87.9) |  | 153 (88.4) | 124 (85.5) |  |
| Female | 41 (12.9) | 20 (13.8) | 21 (12.1) |  | 20 (11.6) | 21 (14.5) |  |
| **Age** |  |  |  | 0.552 |  |  | 0.589 |
| < 65 | 179 (56.3) | 79 (54.5) | 100 (57.8) |  | 95 (54.9) | 84 (57.9) |  |
| ≥ 65 | 139 (43.7) | 66 (45.5) | 73 (42.2) |  | 78 (45.1) | 61 (2.1) |  |
| **Median overall survival (months)** | 135.9 | 69.1 | 158.4 |  | 107.9 | 158.9 |  |
| **(95 % confidence interval)** | (82.7-189.2) | (40.4-97.8) | (124.8-192.0) |  | (42.6-173.2) | (71.8-244.9) |  |
| **Perioperative/ neoadjuvant therapy** |  |  |  | 0.733 |  |  | 0.390 |
| No | 104 (32.7) | 46 (31.7) | 58 (33.5) |  | 53 (30.6) | 51 (35.2) |  |
| Yes | 214 (67.3) | 99 (68.3) | 115 (66.5) |  | 120 (69.4) | 94 (64.8) |  |
| **(y)pT** |  |  |  | 0.589 |  |  | 0.679 |
| 1 | 110 (34.6) | 45 (31.0) | 65 (37.6) |  | 58 (33.5) | 52 (35.9) |  |
| 2 | 58 (18.2) | 28 (19.3) | 30 (17.3) |  | 35 (20.2) | 23 (15.9) |  |
| 3 | 147 (46.2) | 70 (48.3) | 77 (44.5) |  | 79 (45.7) | 68 (46.9) |  |
| 4 | 3 (0.9) | 2 (1.4) | 1 (0.6) |  | 1 (0.6) | 2 (1.4) |  |
| **L** |  |  |  | 0.099 |  |  | 0.636 |
| 0 | 204 (64.2) | 86 (59.3) | 118 (68.2) |  | 113 (65.3) | 91 (62.8) |  |
| 1 | 114 (35.8) | 59 (40.7) | 55 (31.8) |  | 60 (34.7) | 45 (37.2) |  |
| **V** |  |  |  | 0.120 |  |  | 0.563 |
| 0 | 238 (74.8) | 107 (73.8) | 131 (75.7) |  | 133 (76.9) | 105 (72.4) |  |
| 1 | 16 (5.0) | 4 (2.8) | 12 (6.9) |  | 7 (4.0) | 9 (6.2) |  |
| 2 | 64 (20.1) | 34 (23.4) | 30 (17.3) |  | 33 (19.1) | 31 (21.4) |  |
| **Pn** |  |  |  | 0.187 |  |  | 0.753 |
| 0 | 219 (68.9) | 100 (69.0) | 119 (68.8) |  | 120 (69.4) | 99 (68.3) |  |
| 1 | 36 (11.3) | 12 (8.3) | 24 (13.9) |  | 21 (12.1) | 15 (10.3) |  |
| 2 | 63 (19.8) | 33 (22.8) | 30 (17.3) |  | 32 (18.5) | 31 (21.4) |  |
| **G** |  |  |  | 0.849 |  |  | 0.987 |
| 1 | 2 (0.6) | 1 (0.7) | 1 (0.6) |  | 1 (0.6) | 1 (0.7) |  |
| 2 | 68 (21.4) | 28 (19.3) | 40 (23.1) |  | 35 (20.2) | 33 (22.8) |  |
| 3 | 32 (10.1) | 15 (10.3) | 17 (9.8) |  | 17 (9.8) | 15 (10.3) |  |
| 4 | 0 (0.0) | 0 (0.0) | 0 (0.0) |  | 0 (0.0) | 0 (0.0) |  |
| not applicable/unknown | 216 (67.9) | 101 (69.7) | 115 (66.5) |  | 120 (69.4) | 96 (66.2) |  |

**Supplement Table 3**

General clinicopathological values of patients with pT1N0-stage depending on negative or positive SEMA3F as well as NRP2 marker expression. Nr: not reached, NRP2: Neuropilin 2, SEMA3F: Semaphorine 3F. Bold print marks p-values below 0.05.

| **Characteristic** | **Total** | **SEMA3F** | |  | **NRP2** | |  |
| --- | --- | --- | --- | --- | --- | --- | --- |
|  |  | **negative** | **positive** |  | **negative** | **positive** |  |
|  | **n (%)** | **n (%)** | **n (%)** | **p-value** | **n (%)** | **n (%)** | **p-value** |
| **No. of patients** | 110 (100) | 45 (100) | 65 (100) |  | 58 (100) | 52 (100) |  |
| **Sex** |  |  |  | 0.747 |  |  | 0.611 |
| Male | 99 (90.0) | 40 (88.9) | 59 (90.8) |  | 53 (91.4) | 46 (88.5) |  |
| Female | 11 (10.0) | 5 (11.1) | 6 (9.2) |  | 5 (8.6) | 6 (11.5) |  |
| **Age** |  |  |  | 0.249 |  |  | 0.406 |
| < 65 | 61 (55.5) | 22 (48.9) | 39 (60.0) |  | 30 (51.7) | 31 (59.6) |  |
| ≥ 65 | 49 (44.5) | 23 (51.1) | 26 (40.0) |  | 28 (48.3) | 21 (40.4) |  |
| **Median overall survival (months)** | 199.3 | 107.9 | nr |  | 140.9 | nr |  |
| **(95 % confidence interval)** | (112.6-286.0) | (13.9-201.9) | (nr) |  | (77.5-204.4) | (nr) |  |
| **Perioperative/ neoadjuvant therapy** |  |  |  | 0.798 |  |  | 0.449 |
| No | 70 (63.6) | 28 (62.2) | 42 (64.6) |  | 35 (60.3) | 35 (67.3) |  |
| Yes | 40 (36.4) | 17 (37.8) | 23 (35.4) |  | 23 (39.7) | 17 (32.7) |  |
| **L** |  |  |  | 0.526 |  |  | 0.067 |
| 0 | 77 (70.0) | 30 (66.7) | 47 (72.3) |  | 45 (77.6) | 32 (61.5) |  |
| 1 | 33 (30.0) | 15 (33.3) | 18 (27.7) |  | 13 (22.4) | 20 (38.5) |  |
| **V** |  |  |  | 0.667 |  |  | 0.075 |
| 0 | 83 (75.5) | 35 (77.8) | 48 (73.8) |  | 48 (82.8) | 35 (67.3) |  |
| 1 | 1 (0.9) | 0 (0.0) | 1 (1.5) |  | 1 (1.7) | 0 (0.0) |  |
| 2 | 26 (23.6) | 10 (22.2) | 16 (24.6) |  | 9 (15.5) | 17 (32.7) |  |
| **Pn** |  |  |  | 0.917 |  |  | 0.102 |
| 0 | 81 (73.6) | 34 (75.6) | 47 (72.3) |  | 47 (81.0) | 34 (65.4) |  |
| 1 | 3 (2.7) | 1 (2.2) | 2 (3.1) |  | 2 (3.4) | 1 (1.9) |  |
| 2 | 26 (23.6) | 10 (22.2) | 16 (24.6) |  | 9 (15.5) | 17 (32.7) |  |
| **G** |  |  |  | 0.393 |  |  | 0.982 |
| 1 | 2 (1.8) | 1 (2.2) | 1 (1.5) |  | 1 (1.7) | 1 (1.9) |  |
| 2 | 53 (48.2) | 18 (40.0) | 35 (53.8) |  | 27 (46.6) | 26 (50.0) |  |
| 3 | 13 (11.8) | 7 (15.6) | 6 (9.2) |  | 7 (12.1) | 6 (11.5) |  |
| 4 | 0 (0.0) | 0 (0.0) | 0 (0.0) |  | 0 (0.0) | 0 (0.0) |  |
| not applicable/unknown | 42 (38.2) | 19 (42.2) | 23 (35.5) |  | 23 (39.6) | 19 (36.6) |  |

**Supplement Table 4**

Multivariate Cox regression analyses of the pT1N0-3 study cohort. SEMA3F: Semaphorine 3F. Bold print marks p-values below 0.05.

| **Characteristic** | **Borders** | **Hazard Ratio** | **95 % confidence interval** | **p - value** |
| --- | --- | --- | --- | --- |
| **Age** | ≥ 65 vs < 65 | 1.799 | 1.035 - 3.125 | **0.037** |
|  |  |  |  |  |
| **Perioperative/ neoadjuvant therapy** | yes vs no | 1.932 | 1.071 - 3.484 | **0.029** |
|  |  |  |  |  |
| **(y)pN** | ≥ 1 vs 0 | 1.798 | 1.246 - 2.595 | **0.002** |
|  |  |  |  |  |
| **V** | ≥ 1 vs 0 | 0.488 | 0.065 - 3.674 | 0.486 |
|  |  |  |  |  |
| **Pn** | ≥ 1 vs 0 | 1.683 | 0.223 - 12.727 | 0.614 |
|  |  |  |  |  |
| **SEMA3F** | positive vs negative | 0.505 | 0.293 - 0.870 | **0.014** |

**Supplement Table 5**

Multivariate Cox regression analyses of the pT1N0 study cohort. SEMA3F: Semaphorine 3F. Bold print marks p-values below 0.05.

| **Characteristic** | **Borders** | **Hazard Ratio** | **95 % confidence interval** | **p - value** |
| --- | --- | --- | --- | --- |
| **Age** | ≥ 65 vs < 65 | 1.966 | 0.976 - 3.962 | 0.059 |
|  |  |  |  |  |
| **Perioperative/ neoadjuvant therapy** | yes vs no | 2.526 | 1.279 - 4.986 | **0.008** |
|  |  |  |  |  |
| **SEMA3F** | positive vs negative | 0.483 | 0.242 - 0.967 | **0.040** |
